# Stakeholders’ perceptions of personal health data sharing: A scoping review

**DOI:** 10.1371/journal.pdig.0000652

**Published:** 2024-11-20

**Authors:** Prima Alam, Ana Bolio, Leesa Lin, Heidi J. Larson

**Affiliations:** 1 The Vaccine Confidence Project, Department of Infectious Disease Epidemiology, London School of Hygiene & Tropical Medicine, London, United Kingdom; 2 Laboratory of Data Discovery for Health Limited (D24H), Hong Kong Science Park, Hong Kong Special Administrative Region, China; 3 Institute for Health Metrics and Evaluation, University of Washington, Seattle, United States of America; Iran University of Medical Sciences, ISLAMIC REPUBLIC OF IRAN

## Abstract

The rapid advancement of digital health technologies has heightened demand for health data for secondary uses, highlighting the importance of understanding global perspectives on personal information sharing. This article examines stakeholder perceptions and attitudes toward the use of personal health data to improve personalized treatments, interventions, and research. It also identifies barriers and facilitators in health data sharing and pinpoints gaps in current research, aiming to inform ethical practices in healthcare settings that utilize digital technologies. We conducted a scoping review of peer reviewed empirical studies based on data pertaining to perceptions and attitudes towards sharing personal health data. The authors searched three electronic databases–Embase, MEDLINE, and Web of Science–for articles published (2015–2023), using terms relating to health data and perceptions. Thirty-nine articles met the inclusion criteria with sample size ranging from 14 to 29,275. We followed the Preferred Reporting Items for Systematic Reviews and Meta-Analyses extension for Scoping Reviews guidelines for the design and analysis of this study. We synthesized the included articles using narrative analysis. The review captured multiple stakeholder perspectives with an up-to-date range of diverse barriers and facilitators that impact data-sharing behavior. The included studies were primarily cross-sectional and geographically concentrated in high-income settings; often overlooking diverse demographics and broader global health challenges. Most of the included studies were based within North America and Western Europe, with the United States (n = 8) and the United Kingdom (n = 7) representing the most studied countries. Many reviewed studies were published in 2022 (n = 11) and used quantitative methods (n = 23). Twenty-nine studies examined the perspectives of patients and the public while six looked at healthcare professionals, researchers, and experts. Many of the studies we reviewed reported overall positive attitudes about data sharing with variations around sociodemographic factors, motivations for sharing data, type and recipient of data being shared, consent preference, and trust.

## Introduction

### Background

The rapid advancement of health technologies and subsequent growth in sharing of health data for secondary uses has been widely recognized for its potentially transformative role in strengthening health systems, enhancing healthcare outcomes, and improving the efficacy of medical research [1–3]. Personal health data–collected in the form of routine clinical care or medical research, for instance–can be utilized to monitor population health at a local level, identify groups at risk of disease within a country, or measure progress in health and development on a global scale [4]. Evidence of potential breakthroughs include speeding up diagnoses and improving treatment in fields such as cancer, cardiovascular diseases, and rare diseases [3,5–9]. User-generated data collected through wearable consumer devices could help personalize and improve the management of cardiovascular diseases on multiple levels, ultimately resulting in better outcomes on both an individual and a population-wide scale [6]. Furthermore, COVID-19 has acted as a catalyst for digital transformation, accelerating the adoption of digital technologies across various health sectors [10–12]. This surge is largely due to the need for remote operations and services amid social distancing measures and lockdowns. Businesses and healthcare providers swiftly shifted towards online platforms to continue their operations, leading to a rapid integration of digital tools [10]. The pandemic has also spurred innovations in digital health services, such as telemedicine and remote monitoring, highlighting the essential role of technology in managing public health crises [10,11]. This trend in health data sharing has resulted in potentially long-lasting positive effects on medical research and routine health-care delivery [12–14].

Despite potential benefits, evidence reveals concerns around sharing personal information–such as community perceptions about technology use and perceived risks and benefits of sharing data [15–22]. As health technology becomes increasingly pervasive in healthcare, the expanding use of potentially invasive technologies–continuous user-generated data collected through smartwatches and blood glucose monitoring devices, for example–is likely to lead to greater concerns among users, exacerbating existing problems with willingness to use new technologies [23]. For example, Simpson and colleagues’ narrative review highlights trust and privacy concerns as barriers to the sharing of patient-generated data across multiple settings. In addition, healthcare provider perceptions of a technology are likely to affect treatment delivery, especially if it is not considered sufficiently acceptable [21]. Health data are classified as sensitive personal data that require a high safety and security standard [24]. Improving transparency and standardization of data use can affect user perceptions. Therefore, there is a need to follow principles, such as the ‘FAIR Guiding Principles for scientific data management and stewardship’ to ensure data is findable, available, interoperable, and reusable [25].

Given the fast-paced changes within the field of health technology and data generation, this article reviews current literature around various stakeholder–such as the public, patient, healthcare provider, researcher, and policymaker–perspectives on sharing personal health data as well as identify up-to-date barriers and facilitators within the field.

Our article builds on previous reviews that have explored various aspects of health data sharing through an updated search of current evidence in the post-pandemic era. For example, van Panhuis and others described barriers to sharing routinely collected public health data; identifying six categories including motivational, economic, and ethical [4]. Similarly, Husedzinovic and colleagues reviewed the ethical preferences, revealing that patients often preferred a one-time general consent and needed detailed information on privacy protections [17]. A 2018 scoping review looking more broadly at trust in digital health systems identified trust enablers–such as altruism, ease of use, and sociodemographic factors–and impediments–cost, limited accessibility, and fear of data exploitation [26]. Another 2018 review examined the advantages and drawbacks of data sharing, emphasizing the crucial roles of consent and trust [27]. Esmaeilzadeh and Sambasivan (2017) identified seven factors influencing patient attitudes towards health information exchange, including perceived benefits and concerns, and patient characteristics and preferences. In an academic context, Perrier et al. (2020) point out practical challenges to effective data sharing, such as time constraints, resource shortages, lack of skills among researchers, and infrastructure deficits. Given the changing landscape of health technologies, our review provides an opportunity to explore current barriers and facilitators influencing multiple stakeholder perceptions of health data sharing as well as gaps in the research.

### Research objectives

The aim of this article is to review stakeholders’ perceptions and attitudes towards sharing personal data to inform personalized treatments, interventions, and research. We highlight conditions influencing perceptions in the field of health data sharing–such as sociodemographic characteristics, motivational factors, privacy concerns, and trust–as well as gaps in research.

## Methods

We designed and implemented this review using guidelines established by the Preferred Reporting Items for Systematic Reviews and Meta-Analysis Extension for Scoping Reviews (PRISMA-ScR) checklist as presented in S1 Appendix [28]. This review was not registered.

### Search strategy

The authors searched three relevant electronic databases–Embase, MEDLINE, and Web of Science–for peer-reviewed empirical articles using search terms relating to digital health (e.g. mHealth, eHealth, technology, health data, telemedicine, telehealth, mobile applications, smartphone, wearables devices, health information technology, personalized medicine, precision medicine, personal digital assistant, smartphone, big data), perceptions (e.g. perception, trust, confidence, hesitancy), and research type (e.g. qualitative, quantitative, interview, survey). Additionally, we searched citations of identified articles. The authors conducted the search in English and included all articles from 2015 to 2023 in order to incorporate the most relevant and up-to-date evidence from the past decade as this era reflects critical advancements in the development and widespread use of digital health technology. We ran the search on 6 January 2023.

After deduplication using EndNote, two reviewers screened titles and abstracts of the identified records to determine their eligibility based on the predefined inclusion and exclusion criteria. Full-text articles were obtained for all potentially eligible studies and further assessed for inclusion. Any discrepancies between the reviewers were resolved through discussion and consensus, with the involvement of a third reviewer where necessary. The full search strategy for each database is presented in S2 Appendix.

### Inclusion and exclusion criteria

Studies were considered eligible for inclusion if they met the following criteria:

- Peer-reviewed research papers
- Published between 1 Jan 2015 and 1 Jan 2023
- Available in English
- Empirical studies with primary data (quantitative, qualitative, or mixed)
- Address any forms of data sharing such as secondary use of research data already collected, health records, biobank data, big data collected from wearable accessories and other devices or data linkage etc.
- Examine attitudes of research participants, patients, members of the public, healthcare professionals

Studies were excluded if they did not meet these criteria or were opinion pieces, editorials, commentaries, or reviews.

### Data extraction and synthesis

Data extraction was performed by two reviewers using a standardized data extraction form. From each article, we documented the following: author name, year of publication, country of origin, sample size, study design (e.g. qualitative or quantitative), study population, type of data shared, main findings (including barriers and facilitators to data sharing). S3 Appendix provides the coding framework designed by the authors for data extraction.

The authors employed a descriptive and narrative approach to analyze and present the findings from the included studies. This approach involved identifying common themes and patterns across the studies related to the barriers and facilitators of personal health data sharing. We then synthesized the findings and discussed these in relation to the research objectives, considering the perspectives of different stakeholder groups and the global context of personal health data sharing. We identified the barriers and facilitators to data sharing by charting the key issues reported from each study. As part of data extraction, the results section of each study was reviewed to identify various stakeholder priorities, perspectives, expectations, perceptions, and attitudes toward a particular digital health technology or service.

### Quality Appraisal

Given its exploratory nature, the authors did not conduct a formal quality appraisal of the included studies for this scoping review. Nonetheless, the methodological rigor and relevance of each study were considered during data extraction and synthesis to ensure that the review findings were based on credible evidence.

### Conceptual framework

**Fig 1:**
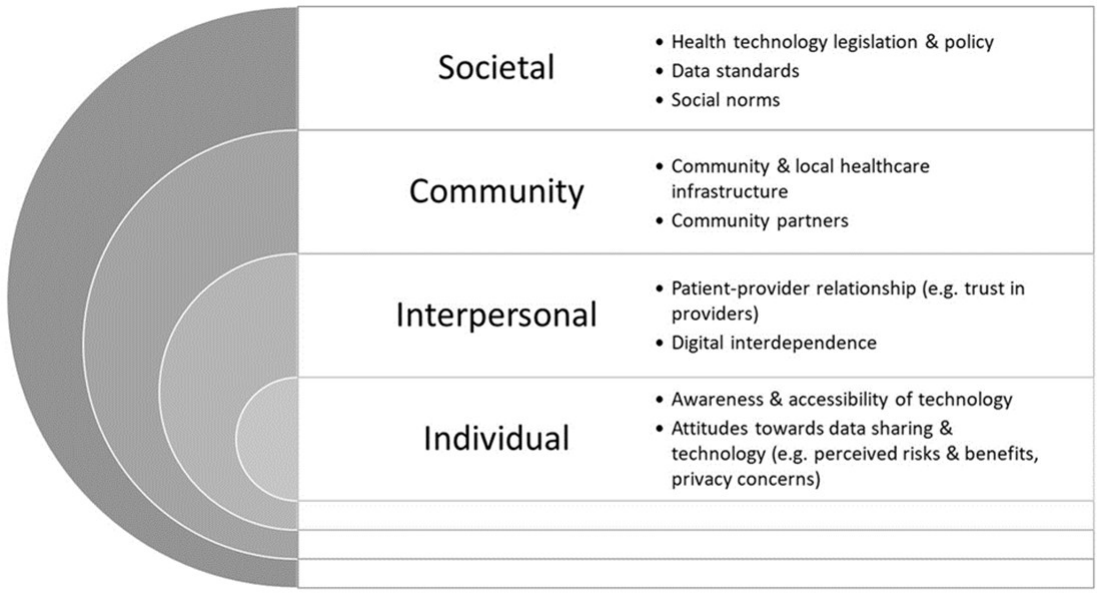
Conceptual framework to identify perceptions of personal health data sharing. Adapted from Esmaeilzadeh and Sambasivan [30] and Richardson, Lawrence [29].

We adapted digital environment elements within the “framework for digital health equity” to create a conceptual framework identifying perceptions of personal health data sharing [29,30]. Our framework–shown below in Fig 1 –incorporates personal, technological, institutional, economic, political, legal, and ethical barriers and facilitators identified in previous reviews [4,26]. The authors utilized this framework to synthesize and present findings from the studies included in this article as this captures key determinants within digital health.

## Results

### Search outcome

**Fig 2:**
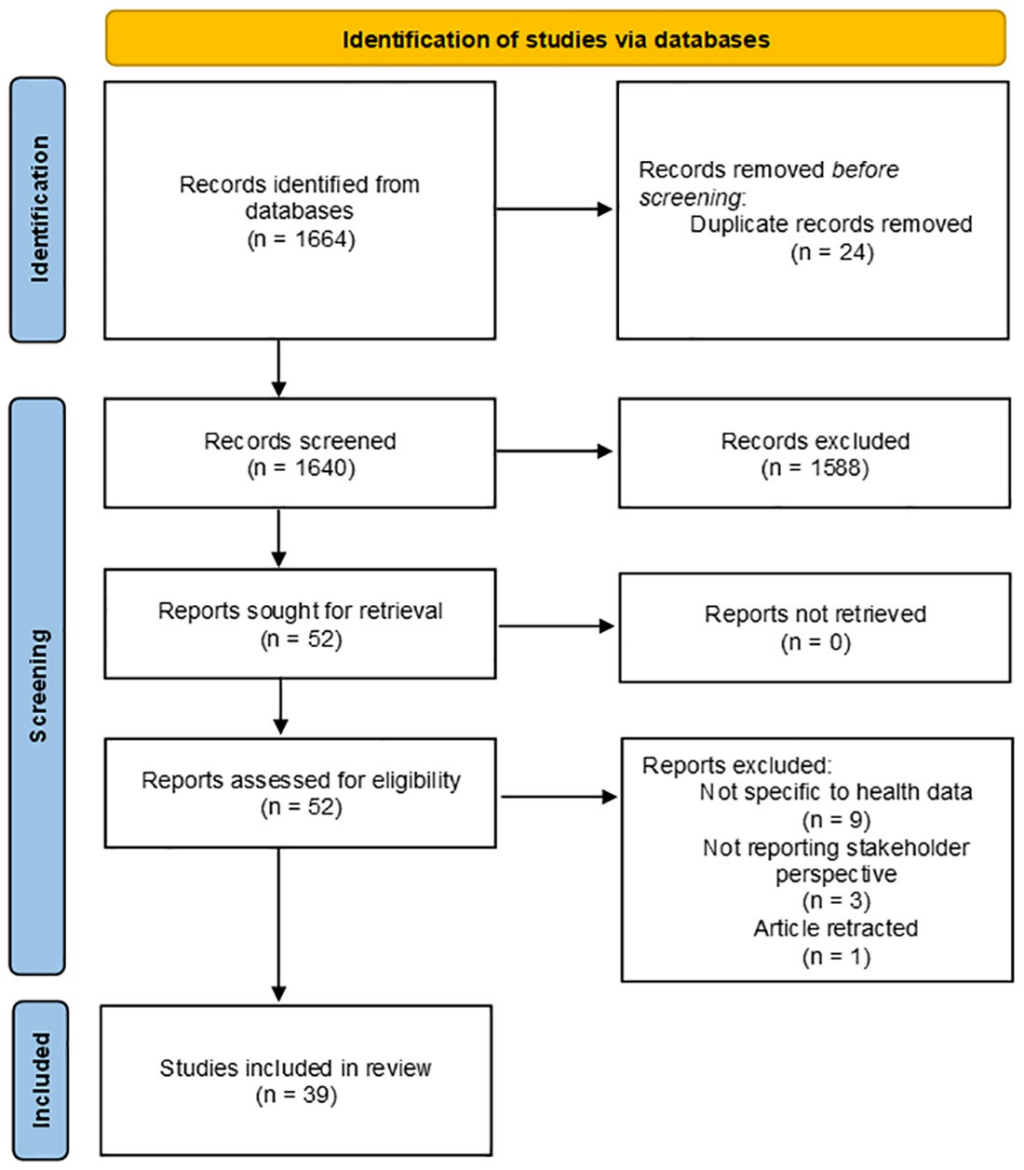
Flow diagram of study selection process.

The authors carried out the search on 6 January 2023 and, after deduplication, we identified 1640 citations. Following title and abstract screening of all citations, PA and AB retrieved 52 articles and included these in the full-text screen. Of these, 39 met the inclusion criteria and were considered in our final analysis (Fig 2). One of the 13 excluded articles were later retracted and, therefore, removed from our review [31].

### Study characteristics

**Fig 3:**
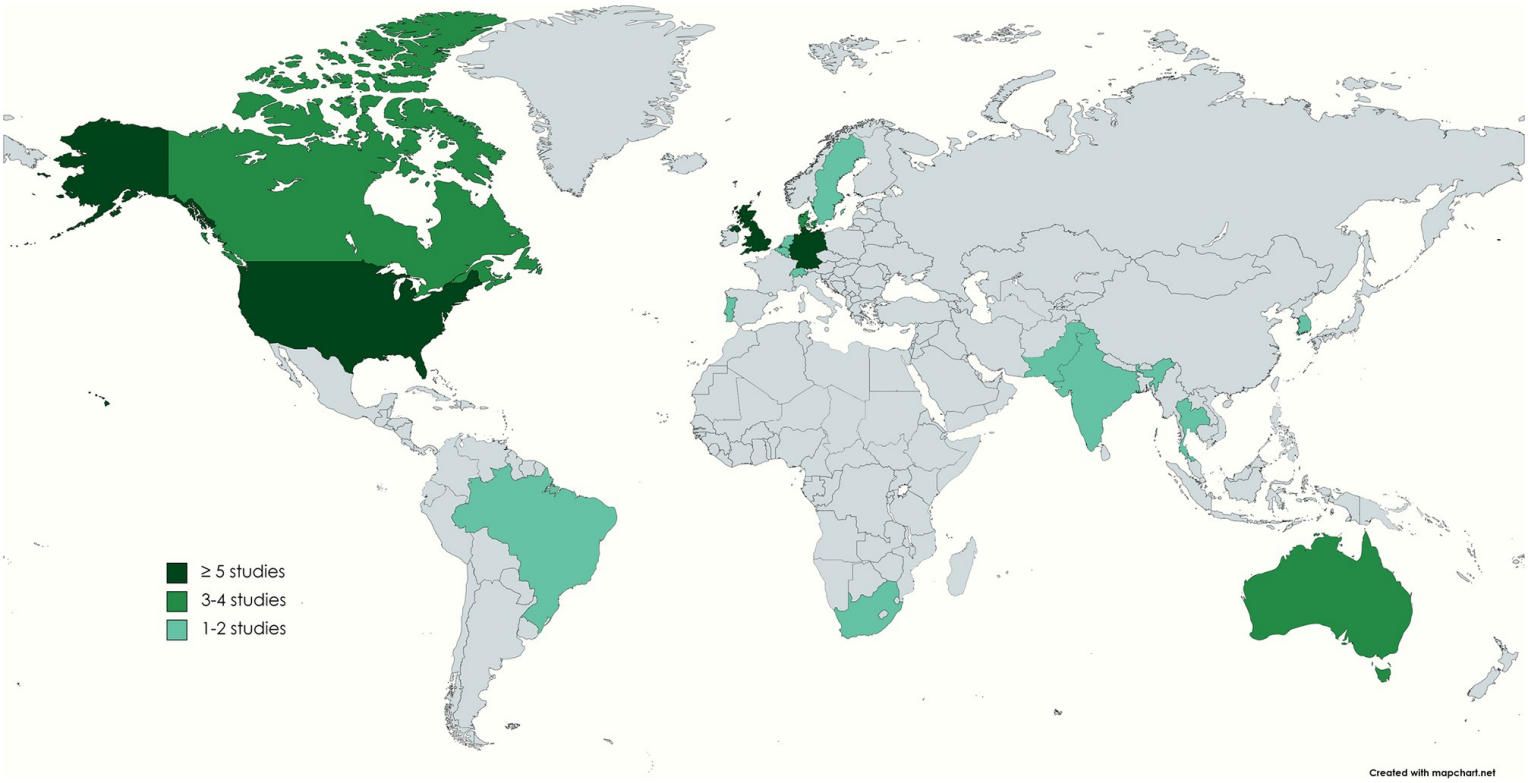
Geographical coverage of included articles on health data sharing perspectives.

The sample size of the 39 included studies sample size ranged from 14 [32] to 29,275 [33]. Many of the studies had a higher percentage of participants over the age of 40 (n = 21) compared with studies including a higher percentage of those below the age of 40 (n = 4). Most of the included studies were based in high-income countries within North America and Western Europe. The top six most studied countries were the United States (n = 8), the United Kingdom (n = 7, including two multi-country studies), Germany (n = 5), Australia (n = 3), Canada (n = 3), and Denmark (n = 3 multi-country studies). Brazil, India, Pakistan, Thailand, and South Africa represented the only low- and middle-income countries included in our review. Fig 3 illustrates the geographical coverage of the included articles.

**Fig 4:**
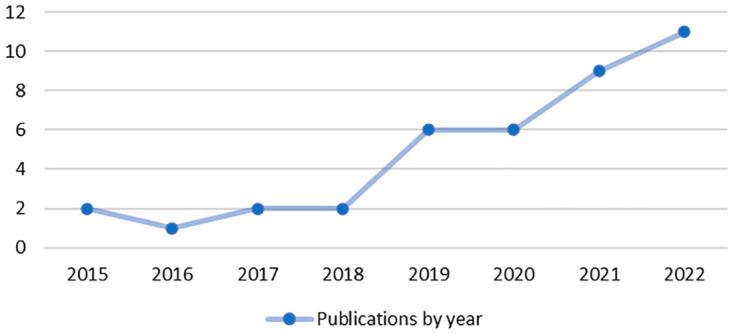
Distribution of publications by year (2015–2022).

Of the 39 articles included, the greatest number was published in 2022 (n = 11, 28%) and 2021 (n = 9, 23%) with only seven articles (18%) published prior to 2019 (Fig 4). The majority of included studies used quantitative methods (n = 23, 59%) and twelve used qualitative methods (n = 12, 31%). The four remaining articles used both quantitative and qualitative methods (10%). All studies except one [34] utilized a cross-sectional design.

**Fig 5:**
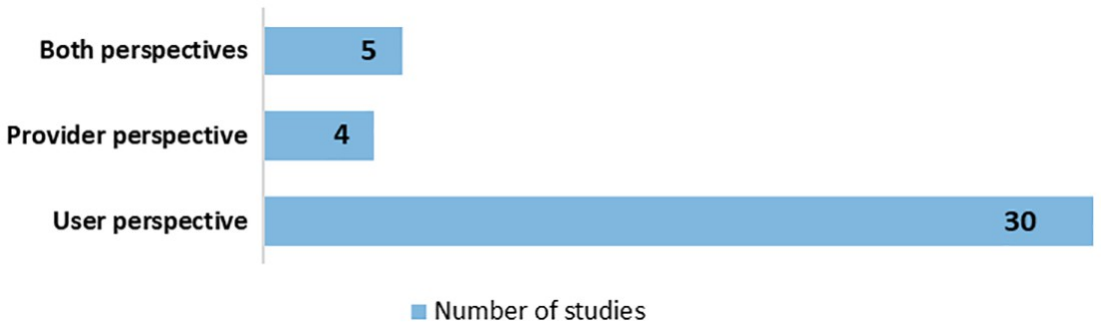
Stakeholder perspectives covered across all included studies.

Most of the included studies (n = 34, 87%) looked at one specific type of stakeholder perspective while the remaining five studies (13%) engaged with multiple stakeholder perspectives in their research. Overall, 29 studies (74%) examined the perspectives of ‘user-side’ stakeholders–including patients and the public. Four studies (10%) looked specifically at ‘provider-side’ perspectives–such as researchers and healthcare practitioners–while a further five studies (13%) incorporated both opinions (Fig 5). The perspectives of patients (n = 15) and the public (n = 13) were the most studied followed by healthcare professionals, researchers, scientists, and experts (n = 11). Eighteen studies focused on health conditions such as cancer (n = 4), mental health (n = 4), chronic health conditions (n = 4) or rare diseases (n = 2).

**Fig 6:**
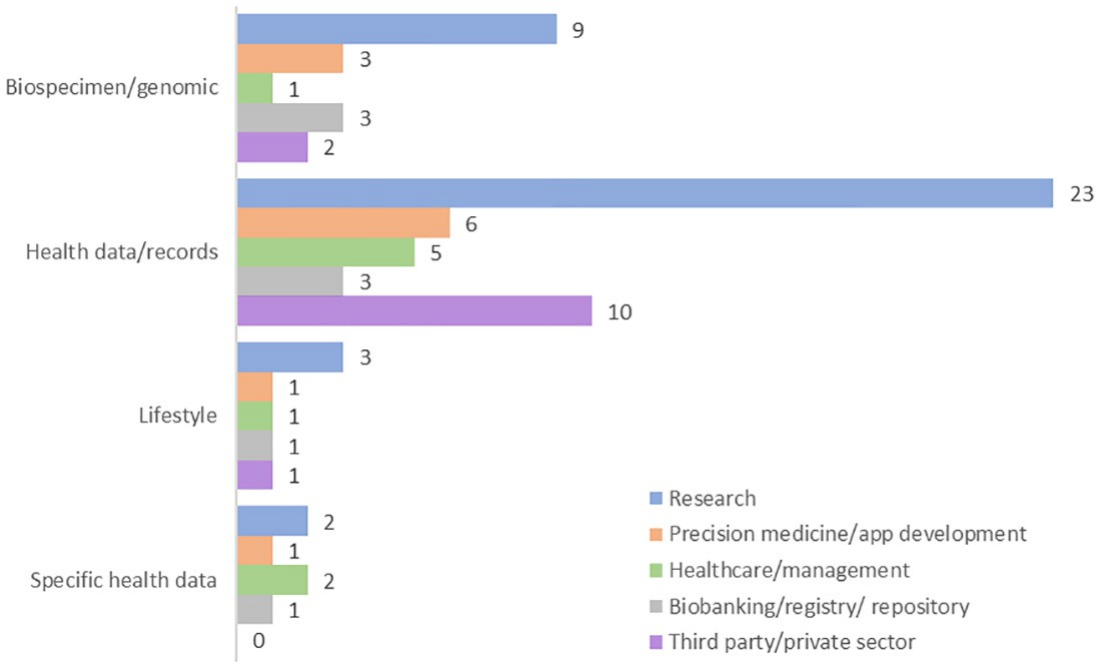
Trends in type of health data shared by use of data shared.

Most studies included information on health data and records (n = 33, 66%) and/or biospecimen or genomic data (n = 10, 19%). In terms of the purpose of shared data, many of the studies focused on secondary data use for research (n = 26, 50%) and/or the private sector (n = 10, 19%). Fig 6 summarizes the type and use of data sharing as reported by included studies.

Tables 1 and 2 below summarize study characteristics as well as type and recipient/purpose of data being shared across all 39 included studies.

**Table 1:** Summary of study characteristics for included articles.

First author, year	Country	Topic	Type of health data	Health condition	Research design	Methods	Study population	Age ^a^	Sample size
Cheah, 2015	Thailand	Stakeholders’ attitudes and experiences about good data sharing practice	Health data for clinical research	-	Qualitative	- Interview - Focus group	- Researchers - Community representatives	Not Reported	25
Sheikh, 2017	- Pakistan - Denmark	Unpacking trust within research participation	Blood sample and health data for genomic research	-	Qualitative	Interview	Research participants	Not Reported	48
Hate, 2015	India	Ethical data sharing practice in research involving women and children in urban India	Demographic/ household details, images/videos, and medical records for research	-	Qualitative	- Semi-structured interview - Focus group	- Researchers - Research participants	Not Reported	66
Staunton, 2021	South Africa	Stakeholder perspectives on protection of personal health information	Health data for research	-	Qualitative	Semi-structured Interview	- Doctors - Scientists - Government representatives	Not Reported	19
Barnes, 2020	Canada	Patient perspectives on biobanking	Genomic and personalized health data for research	Stroke	Quantitative	Survey (electronic)	Patients	47% 69	196
Wiesner, 2018	Germany	Motivational and privacy aspects of wearable technology used by active citizens	Activity health data	-	Quantitative	Survey	Runners	≥*16*	845
Vilaza, 2021	- Denmark - Brazil	Public attitudes towards digital health repositories	Health data for digital health research repository	-	Quantitative	Survey	Public	58% 18–27 29% 28–37	1600
Kim, 2020	South Korea	Public attitudes towards precision medicine	Clinical data, specimens, genetic data, environmental and lifelog data for research and precision medicine	-	Quantitative	Survey (online)	Public	26% 50–59 20% 40–49 19% ≥60	1500
Lysaght, 2021	Singapore	Public trust in data sharing for precision medicine	Health data for precision medicine	-	Quantitative	Survey	Public	40% 40–59 33% 21–39 27% ≥60	1000
Nwebonyi, 2022	Portugal	Public views on health data sharing, access and (re)use	Medical records, genetic and phenotypic data for research and development	Rare diseases	Quantitative	Survey	- Patients - Informal carers	58% <18 (patients) 87% >30 (carers)	651
Braunack-Mayer, 2021	Australia	Public attitudes of sharing government health data with private sector	Government health data for research and development	-	Quantitative	Survey (online)	Public	20% <29 35% 30–49 25% 50–64 18% ≥65	2537
Zhang, 2021	USA	Privacy concerns and data value of mobile mental health systems	Mental health data to improve mental illness management	Mental health	Quantitative	Survey (online)	Members of online mental health communities	42% 26–30 28% 18–25 25% 31–35	170
Zhang, 2022	USA	Public attitudes towards pharmacogenomic testing and statewide database	Pharmacogenomic data for clinical and research purposes	-	Quantitative	Survey (electronic)	Public	Median: 42–53	808
Young, 2022	USA	Expert perspective on data elements in routine care of patients	Physical function in medical records data to improve care	-	Mixed	- Focus group - Longitudinal eDelphi survey	- Healthcare experts - Patients	Mean: 24 (experts) ≥65 (patients)	26
Wetzels, 2018	Netherlands	Patient perspectives on health data privacy and management	Health and lifestyle data for healthcare	Cardiac condition	Qualitative	Focus group	Patients	Mean: 67	23+5 FGDs
Gotzl, 2022	Germany	Stakeholder perspectives on mobile mental health apps for young people	Personal data to inform mental health app	Mental health	Mixed	- Focus group - Survey	- Young adults - Experts	16–25	667+16 FGDs
Hutchings, 2022	Australia	Patients attitudes towards re-use of administrative and clinical trial data	Health and clinical trial data to improve patient care	Breast cancer	Quantitative	-Survey - Interview	Patients	43% >65 34% 55–64 23% <54	132
Hartmann, 2019	Germany	Attitudes towards mobile app to self-monitor and manage depression	Health data to inform mobile app	Mental health (depression)	Quantitative	Survey (online)	Patients	Mean: 38	998
Trinidad, 2020	USA	Public comfort with sharing health data with third-party commercial companies	Health data sharing with third-party commercial companies	-	Quantitative	Survey	Public	31% >60 12% 18–29	1841
Tosoni, 2022	Canada	Patient consent preferences on sharing personal health information during COVID-19	Health data for research and commercial purpose	Cancer	Mixed	- Survey - Focus group	Patients	Pre-, post-Covid 59%, 76% 50–74	417
Tosoni, 2019	Canada	Patient consent preferences on sharing personal health information with academic healthcare institution	Health data for research	Cancer	Quantitative	Survey	Patients	59% 50–74	222
Summers, 2022	UK	Public willingness to share data in the context of COVID-19	Health data for healthcare services	Chronic health conditions	Mixed	Survey (online)	Public with chronic health conditions	65% 55–74	4764
Spencer, 2016	UK	Patient perspectives on the use of anonymized health data for research	Health data for research	Chronic health conditions	Qualitative	- Interview - Focus group	Patients	Mean: 61	40
Pletscher, 2022	Switzerland	Public willingness to share anonymized routinely collected clinical health data	Health data for research	Chronic health conditions	Quantitative	Survey (online)	- Public - Patients	Public, patients: 35%, 21% 18–39 43%, 49% 40–64 22%, 30% >64	1231
Hassan, 2020	UK	Public attitudes towards sharing genomic data within NHS	Genomic data for clinical care	-	Qualitative	Focus group	- Patients and family - Public	- 16–18 (patients) - ≥18 (public)	44
O’Brien, 2019	USA	Patient perspectives on linkage of health data	Health data for research	Multiple conditions	Quantitative	Survey (online)	Patients	69% 40–65	3516
Ostherr, 2017	USA	Trust and privacy in the context of user-generated health data	Health data used outside clinical settings	-	Qualitative	Semi-structured interview	- Researchers - Health startups - Public	Not Reported	32
Ivanova, 2020	USA	Mental health professionals’ views on patient-controlled data sharing	Health records for consent	Mental health	Qualitative	Semi-structured interview	Healthcare professionals (HCP)	Not Reported	20
Johansson, 2021	- UK - Norway - Iceland - Sweden	Public preferences for health data sharing	Digital health data for policymaking	-	Quantitative	- Discrete choice experiment	Public	Mean: 48–50	1967
Shah, 2019	- Denmark - Sweden - Netherlands - UK	Participants’ views about data governance post-project	Health data for post-project sharing	Diabetes	Quantitative	Survey	Research participants	73% ≥61	855
Schults, 2019	Australia	Healthcare practitioner perspectives and experiences of vascular access device data	Vascular access data for clinical quality registry	-	Qualitative	Semi-structured interview	Healthcare practitioners	Not Reported	19
Richter, 2021	Germany	Patient attitude towards data-donation for medical research	Health data for medical research	-	Quantitative	Survey (online)	Patients	- Survey 1,2: 51–65 29%, 36% (men) 32%, 34% (women)	508
Amorim, 2022	Portugal	Perceived benefits and risks of sharing genomic data for research	Genomic data for clinical research	Rare diseases	Quantitative	Survey (online)	- Patients - Informal carers - HCP	56% <18 (patients) 75% 30–49 (carers) 54% >49 (HCP)	700
Brall, 2021	Switzerland	Public willingness to participate in personalized health research and biobanking	Health data and biological samples for health research and biobanking	-	Quantitative	Survey (online)	Public	20% 55–64 18% 45–54 17% 65-74^b^	5086
Broes, 2020	Belgium	Patients’ attitudes towards re-use of clinical trial samples and data	Health data and blood samples and tumor tissue	Cancer	Qualitative	Interview	Patients	Not Reported	16
Brown, 2022	UK	Patients’ attitudes and experiences data sharing	Health and lifestyle data	Chronic health conditions	Qualitative	- Interview - Card sorting	Patients	Mean: 42	14
Buhr, 2022	Germany	Public attitudes towards data sharing through mobile apps for pandemic research	Test results, contact tracing data, fitness data for research	-	Quantitative	Survey (phone)	Public	20% 50–59 19% 40–49 18% ≥70 17% 18–30	924
Jones, 2021	UK	Public opinion on data sharing preferences	Mental and physical health data for research	-	Quantitative	Survey (online)	- Patients - Carers - Public	23% 55–65 22% 45-54^b^	29,275
Kim, 2019	USA	Patient perspectives on sharing medical data and biospecimens for research	Health records and biospecimens for research	-	Quantitative	Survey	Patients	Mean: 51	1246

a: Mean/median age or age range as reported in included articles and rounded up to nearest percentage; a: Percentages calculated from reported proportions by authors.

**Table 2:** Type and use of health data investigated across included studies.

First author, year	Type of health data				Use of health data					
	Biospecimen/ genomic	Health data and records	Lifestyle	Specific health data	Research	Precision medicine/app development	Healthcare services/ patient care	Health management	Biobanking/ registry/ repository	Third party/ private sector
Cheah, 2015	No	Yes	No	No	Yes ^a^	No	No	No	No	No
Sheikh, 2017	Yes	Yes	No	No	Yes	No	No	No	No	No
Hate, 2015	No	Yes	No	No	No	No	No	No	No	No
Staunton, 2021	No	Yes	No	No	Yes	No	No	No	No	No
Barnes, 2020	Yes	Yes	No	No	Yes	No	No	No	Yes	Yes
Wiesner, 2018	No	No	Yes	No	No	No	No	No	No	No
Vilaza, 2021	No	Yes	No	No	Yes	No	No	No	Yes	No
Kim, 2020	Yes	Yes	Yes	No	Yes	Yes	No	No	No	No
Lysaght, 2021	No	Yes	No	No	No	Yes	No	No	No	Yes
Nwebonyi, 2022	Yes	Yes	No	No	Yes	Yes	No	No	No	No
Braunack-Mayer, 2021	No	Yes	No	No	Yes	Yes	No	No	No	Yes
Zhang, 2021	No	Yes	No	Yes	No	No	No	Yes	No	No
Zhang, 2022	Yes	No	No	No	Yes	No	No	No	No	No
Young, 2022	No	Yes	No	No	No	No	Yes	No	No	No
Wetzels, 2018	No	Yes	Yes	No	No	No	Yes	No	No	Yes
Gotzl, 2022	No	Yes	No	Yes ^b^	No	Yes	No	No	No	No
Hutchings, 2022	No	Yes	No	Yes ^c^	No	No	Yes	No	No	No
Hartmann, 2019	No	Yes	No	No	No	Yes	No	No	No	Yes
Trinidad, 2020	No	Yes	No	No	No	No	No	No	No	Yes
Tosoni, 2022	No	Yes	No	No	Yes	No	No	No	No	Yes
Tosoni, 2019	No	Yes	No	No	Yes	No	No	No	No	No
Summers, 2022	No	Yes	No	No	No	No	Yes	No	No	No
Spencer, 2016	No	Yes	No	No	Yes	No	No	No	No	No
Pletscher, 2022	No	Yes	No	No	Yes	No	No	No	No	No
Hassan, 2020	Yes	No	No	No	No	No	Yes	No	No	No
O’Brien, 2019	No	Yes	No	No	Yes	No	No	No	No	No
Ostherr, 2017	No	Yes	No	No	Yes ^d^	No	No	No	No	Yes
Ivanova, 2020	No	Yes	No	No	Yes ^e^	No	No	No	No	No
Johansson, 2021	No	Yes	No	No	Yes ^f^	No	No	No	No	No
Shah, 2019	No	Yes	No	No	Yes ^g^	No	No	No	No	No
Schults, 2019	No	No	No	Yes ^h^	No	No	No	No	Yes	No
Richter, 2021	No	Yes	No	No	Yes ^g^	No	No	No	No	Yes
Amorim, 2022	Yes	No	No	No	Yes	No	No	No	No	No
Brall, 2021	Yes	Yes	No	No	Yes	No	No	No	Yes	No
Broes, 2020	Yes	Yes	No	No	Yes	No	No	No	No	No
Brown, 2022	No	Yes	Yes	No	Yes	No	No	No	No	No
Buhr, 2022	No	No	Yes	Yes ^i^	Yes	No	No	No	No	No
Jones, 2021	No	Yes	No	Yes ^j^	Yes	No	No	No	No	No
Kim, 2019	Yes	Yes	No	No	Yes	No	No	No	No	Yes

^a^: Clinical research;

^b^: Data around mental health;

^c^: Clinical trial data;

^d^: Research outside clinical settings;

^e^: Research on consent;

^f^: Research to inform policymaking;

^g^: Including secondary data sharing;

^h^: Vascular access data;

^i^: COVID-19 test results and contact tracing information;

^j^: Mental and physical health data.

### Perspectives on data sharing

Most of the studies we reviewed reported overall positive stakeholder attitudes about data sharing with certain factors impeding or facilitating willingness to share. Across the studies, we identified variations in perspectives on data sharing based on sociodemographic characteristics, motivational factors, ethical and privacy concerns, and differing levels of trust. Table 3 highlights the key factors we identified through narrative analysis as well as the level of influence, as outlined earlier in our conceptual framework.

**Table 3:** Summary of factors influencing stakeholders’ perceptions of sharing personal health data identified in included studies.

Level of influence^a^		Factors	Description & references
Individual		**Sociodemographic characteristics** ^b^	
		Age	- Variations between age groups: both younger age groups^c^ [35–38] and older age groups willing to share data [39–41]
		Gender	- Variations by gender: men more likely to share data in different settings [39,42]
		Education	- Variations between education level: more educated more willing to support data sharing for health [35–39,43]
		**Motivational factors for data sharing**	
		Altruism	- Willingness to share data when this is perceived to help others [36,44–48]
		Health conditions	- Willingness to share data when this is perceived to help with personal health condition [32,44,49,50]
		COVID-19 experiences	- Willingness to share data increased during/post COVID [51–53]
	Interpersonal	**Ethics and privacy concerns**	
		Consent preference	- No consensus on best approach to obtain consent but general support across stakeholder groups for one-time consent over explicit consent with some concerns [47,50,54–56]
		Privacy concerns	- Main privacy concerns expressed by all stakeholder groups included lack of transparency, patient rights, privacy breaches, misuse of sensitive and identifiable data, and discrimination against individuals with stigmatized diseases [36,39,47,52,54,56–58]
		**Levels of trust**	
		Trust in providers	- High levels of institutional trust (e.g. sharing data with government) [44,48,53,55]
		Type of data shared	- Less willingness to share identifiable data than anonymized data, and lifestyle data than medical data [47,49–51,58,59]
		Recipient of data shared	- More willingness to share data with research institutions and universities than commercial, private, or third-party entities according to multiple stakeholders [40,49,52,55,59,60]

^a^ Individual and interpersonal levels of influence overlap for ethics and privacy concerns;

^b^ Based on data reported for public/patients;

^c^ As defined and reported by the authors of the studies included in this review.

### Sociodemographic characteristics

Results varied across the studies that analyzed the association between sociodemographic characteristics and willingness to share personal health data. Age, education, and gender were among the key sociodemographic characteristics associated with personal health data sharing attitudes. For example, six studies found that more educated participants were more willing to share data [35–39,43], while seven studies reported significant variations by age [35–41] and two by gender [39,42]. It is noteworthy that most of the studies incorporating provider perspectives–such as those of researchers and physicians–did not provide sociodemographic delineations for their participants. For example, two qualitative studies of researchers in Thailand and the US did not report the age of participants [54,61].

### Motivational factors

Participants across several studies stated that altruistic benefits of data sharing health outweighed the risks. All stakeholders suggested identified helping others, helping future patients, discovering a cure for untreatable disease or development of effective treatments, and promoting scientific progress as motivators for sharing personal health data [36,43,45,46,48,54,55].

Furthermore, participants with health conditions were supportive of sharing their own health data to improve treatments. For example, nearly 98% of stroke patients in a Canadian study said they would be willing to provide a blood sample to help develop a blood test for stroke. In another multi-country study, 97% of diabetic patients interviewed were supportive of their data being shared for secondary use [60]. O’Brien and colleagues reported that 94% of patients in their US-based study were willing to share health data to help their doctors make better decisions and make new therapies available faster [62].

Experiences of the COVID-19 pandemic also appeared to be a motivating factor for sharing personal health data. A significant proportion of people felt that their own attitudes had shifted due to the COVID-19 pandemic. More people reported being comfortable with sharing private health data with any organization during rather than before the COVID-19 pandemic. More people reported being comfortable with sharing anonymized data than personally identifiable data. Around 67% disagreed or strongly disagreed with sharing their private health data without anonymization [51]. Willingness to share data also varied depending on who the data would be shared with (e.g. government, researchers, health system), highlighting trust as a key determining factor regarding who may have access to shared personal health data and how it may be used in the future [51].

Three studies–based in Canada, the UK, and Germany–looked specifically at the impact of COVID-19 as well as the use of pandemic apps and public willingness to share personal health data [51–53]. More participants reported being comfortable with sharing personal health data during the COVID-19 pandemic rather than before [51]. A significant proportion of people felt that their own attitudes had shifted as a result of the pandemic with more people reported being comfortable with sharing private health data with any organization during rather than before the pandemic.

A Canadian study of cancer patients found that during the pandemic, patients were more comfortable sharing data with all parties (90% vs 79%, p = 0.009), except with commercial entities [52]. In a Germany study exploring the use of pandemic apps, an overwhelming majority (84%) of smartphone users were willing to provide their app data for state-funded research and almost all app users (97%) stated they were willing to share data, while 74% of nonusers supported data sharing via an app [53].

On the other hand, there were concerns around privacy and use of identifiable health data during COVID-19. A UK-based study of chronic health patients found that, post-COVID, almost half of respondents were concerned or very concerned about who would have access to their personal health data in the context of the pandemic and how their personal health data may be used in the future [51].

### Ethical and privacy concerns

Of the studies that explored ethics and consent preferences, there was more overall acceptance rather than opposition for sharing data without explicit consent from both users and providers. For example, 76% of participants in a UK study were willing to share data without explicit consent versus 20% who opposed this [33]. Similarly, a Singapore study reported 64% of participants were willing to share de-identified health data with institutions without consent for each study [55]. At the same time, Lysaght and others concluded that the users and uses of data influenced public trust and willingness to share data than either the sensitivity of the data or the consent procedures in Singapore [55]. In another study surveying runners in Germany, 42% stated that they were not concerned if data might be shared without their consent while 35% would not accept sharing data without their consent [42]. At the same time, transparency was strongly desired, particularly with commercialization of data being shared [48,52,56]. Transparency of data used as well as better understanding of data protection was also highlighted by healthcare providers and scientists in a South African study [63].

Participants across various studies predominantly expressed concerns over potential misuse of data, lack of transparency in the process, and sharing of identifiable and sensitive data [36,39,47,52,54,56–58]. For example, participants in a multi-country study were very concerned their data being used in unethical projects (76%), profit making without consent (69%), and cyberattacks (66%) [47]. Researchers and healthcare professionals in Thailand, India, South Africa, and Portugal expressed their concern over data protection and the potential risk of data breaches [45,54,56,63]. The Indian study also highlighted participants’ skepticism around the use of data to harm participants or meet vested interests [56]. Another study examining public comfort with sharing health data with third-party commercial companies revealed that as privacy concerns increased, comfort with sharing health data with third-party commercial companies decreased [38].

### Trust

"I’m generally quite trustful of hospitals and GPs [general practitioners]"

- Interviewee [48]

Several included studies investigated the role of trust in data sharing preferences. Overall trust in governmental/public, or government-funded research institutes and organizations was much higher than trust in private organizations–such as private clinics and health insurance companies [40,49,52,53,55,59,60]. On the other hand, researchers in a US-based study perceived that individuals were more resistant to sharing health data for scientific studies compared with companies that sold the devices and apps they used [61]. Overall, there was significant distrust of private health data use by social media platforms (e.g. Facebook and Twitter). For example, Zhang and others reported that most respondents in their US-study trusted health professionals (78%) and researchers (73%) to keep their data private [37]. A 2021 Singapore study also found that respondents most trusted public health institutions and hospitals and that Facebook was the least trusted institution [55]. Most patients and carers surveyed in a Portuguese study perceived trust in research institutions and trust in research teams as very important issues when making decisions about sharing data [64]. Furthermore, participants in the same study who considered trust in research institutions as very important rated higher the importance of being involved in decisions about data sharing, data access, data use, and data reuse [64].

While most studies indicated that people trust their data with the government and health organizations, two studies based in India and South Africa found that respondents were skeptical about sharing personal health data [56,63]. For instance, Staunton and colleagues reported that historical exploitative research, inequitable collaborations, and historical use of biological data has resulted in resistance among many in South Africa to the sharing of personal health data [63].

Types of recipients that participants trusted to share their personal health data with also depended on the type of data being shared. For instance, a study of the German running community found that runners preferred to exchange tracked data with recipients they trusted such as friends (52%), family members (43%), or a physician (32%) [42]. However, another German study surveying mental health patients suggested a low preference (13%) for sharing personal health data with friends [65].

“It is very unclear how the commercial side of health care remains separated from the actual care with these systems.”

- Patient [59]

Most studies that explored the purpose of data being shared found that respondents had a low preference towards sharing health data with insurance companies [49,55,65]. For example, patients interviewed in a Canadian study suggested that they were less supportive of data sharing if a commercial entity was the recipient of the health information (53%) compared with nonprofit organizations such as universities (87%) [49]. Two study showed participants were ambivalent about sharing their health data with commercial actors [61,66].

## Discussion

Most studies included in this review reported generally positive perspectives around data sharing from different stakeholders with participants identifying altruism and the development of effective treatments as key motivators for sharing personal health data. Overall, findings from our review suggest relatively higher trust in public or government-funded research institutes and organizations than in private organizations–such as insurance companies, social media companies, and other commercial companies. This is in line with existing global evidence towards widespread general support for data sharing for research purposes [2,67]. Despite evidence of predominantly supportive views towards data sharing, studies also noted concerns around data privacy through data breaches and misuse of data, a lack of trust in commercial use of data, and skepticism around the supposed benefits of data sharing [68].

### Research gaps and further research

Our review suggests research on health data sharing in low- and middle-income countries is markedly limited. Data sharing research was geographically concentrated in North America and Western Europe, as previous reviews have illustrated [67]. As a result, there is also a lack of analysis that considers diverse demographic characteristics. Moreover, the majority of studies are cross-sectional, with an absence of standardized studies that span multiple countries or emphasize changes over time. Further comparable research across diverse settings is required to build on current evidence, especially to understand variations between data sharing within high-income countries and low- and middle-income countries (LMICs) [69].

Much of the existing research centers on individual perspectives, primarily examining the views of existing patients. Of the nine studies that engaged with ‘providers’–including healthcare providers, researchers or experts–most did not contextualize or position perceptions from a patient/public-provider relational viewpoint. This leaves out broader layers of analysis, like the interpersonal, community, and societal levels of the digital environment and trust. Key areas–such as technology biases, healthcare infrastructure challenges, and policy implications related to technology–are often sidelined, as highlighted by Richardson et al. (2022). This research gap becomes even more pertinent given that reviewed studies identified institutional trust as a key factor influencing attitudes towards data sharing. How does trust play out across broader healthcare settings and what are some intersecting systemic concerns, for example?

Another similar gap in the research relates to variations in access across different regions, in-country settings, and across sociodemographic groups. As the scope of the included studies did not extend to investigating attitudinal variations across subgroups, none of the studies examine how access to technology may impact attitudes towards equitable data sharing [69]. How would trends in adoption and access to technology–such as limited use of mobile phones by women in rural central India, for example [70]–be reflected in attitudes towards data sharing? In other words, what type of data is being collected, from whom, at what cost, and for whose benefit? There is an absence of comprehensive cost-benefit analyses, signaling an area ripe for further investigation.

There is an urgent need to address the challenges LMICs face when trying to integrate digital health technologies [24]. Further research is essential to address challenges ranging from the need for supportive environments and resources, to infrastructure development for digital transitions, and to improvements in education and capacity building [24,69]. Other challenges to address include internet connectivity, updating older infrastructure, navigating technology ownership issues, and handling concerns related to privacy, security, and the application of global standards [24]. For example, routine health information data represent an underused source of data and could be made more available and further embraced by the research community in LMIC health systems [71].

The rapid rate of development in precision medicines highlights that staying current with research in digital health is essential. There is a pressing need to gather globally comparable evidence that delves into perspectives and attitudes regarding personal health data sharing, especially in low- and middle-income settings. Achieving this would not only aid in standardizing ethical practices but also in documenting global trends. Furthermore, engaging in longitudinal research that examines viewpoints before and after data sharing would be instrumental in figuring out if and how attitudes shift over time. It is also crucial to conduct more detailed qualitative research that looks into the barriers and facilitators of data sharing at every level—from individual to interpersonal, community, and even societal scales. To truly understand the nuances, it is imperative to gather data across varied sociodemographic markers for all stakeholders, emphasizing age and education. Analyzing this data would help in segmenting populations and diving deeper into the varying perspectives on sharing personal health data. An interesting aspect to consider is observing the differences in healthcare delivery systems and understanding their influence on perceptions about data sharing [72]. Such studies can address the lack of evidence around diverse demographics as well as broader global health challenges.

### Strengths and limitations

As with any kind of research, our article had both strengths and limitations worth mentioning. This review captured multiple stakeholders’ perspectives on personal health data sharing across various contexts, with a diverse range of barriers and facilitators that impact data-sharing behavior. We searched multiple electronic databases and included both quantitative and qualitative studies. Our research also builds on previous systematic and scoping reviews to contribute towards better understanding the dynamic field of data sharing. Despite these strengths, our review had several limitations. Firstly, the search was restricted to articles published in English, which may have led to the exclusion of relevant studies in other languages. Secondly, the scope of this review focused on perceptions of personal health data sharing. As such, our search strategy may not have captured other relevant information such as health legislation and policies. Thirdly, the narrative synthesis approach used in this review–while effective in identifying common themes and guided by a conceptual framework–may be subject to reviewer bias in the interpretation of the findings. Finally, as this was a scoping review, a formal quality appraisal of the included studies was not conducted.

## Supporting information

S1 Appendix PRISMA 2020 checklist. (DOCX)

S2 Appendix Full search strategy. (DOCX)

S3 Appendix Coding framework for data extraction. (DOCX)
