# Supplementary material for: Stakeholders’ perceptions of personal health data sharing: A scoping review
Source: PLOS Digit Health. 2024 Nov 20;3(11):e0000652. doi: 10.1371/journal.pdig.0000652 (PMC11578505; doi:10.1371/journal.pdig.0000652)
Supplement: S2 Appendix — (DOCX) [file pdig.0000652.s002.docx]

**S2 Appendix: Full search strategy**

**MEDLINE**

**1** (patient* or public or citizen* or lay).mp. [mp=title, book title, abstract, original title, name of substance word, subject heading word, floating sub-heading word, keyword heading word, organism supplementary concept word, protocol supplementary concept word, rare disease supplementary concept word, unique identifier, synonyms]

**2** (attitude* or view* or perspective* or opinion* or trust * or mistrust).mp. [mp=title, book title, abstract, original title, name of substance word, subject heading word, floating sub-heading word, keyword heading word, organism supplementary concept word, protocol supplementary concept word, rare disease supplementary concept word, unique identifier, synonyms]

**3** ("data shar*" or "data access" or "data transfer").mp. [mp=title, book title, abstract, original title, name of substance word, subject heading word, floating sub-heading word, keyword heading word, organism supplementary concept word, protocol supplementary concept word, rare disease supplementary concept word, unique identifier, synonyms]

**4** *research/ or "precision medicine".mp. or "tailored medicine".mp. or "personal* medicine".mp. [mp=title, book title, abstract, original title, name of substance word, subject heading word, floating sub-heading word, keyword heading word, organism supplementary concept word, protocol supplementary concept word, rare disease supplementary concept word, unique identifier, synonyms]

**5** 1 and 2 and 3 and 4

**6** limit 5 to (english language and yr="2012 -Current")

**Embase**

**1** (patient* or public or citizen* or lay).mp. [mp=title, abstract, heading word, drug trade name, original title, device manufacturer, drug manufacturer, device trade name, keyword heading word, floating subheading word, candidate term word]

**2** (attitude* or view* or perspective* or opinion* or trust).mp. or *trust/ [mp=title, abstract, heading word, drug trade name, original title, device manufacturer, drug manufacturer, device trade name, keyword heading word, floating subheading word, candidate term word]

**3** ("data shar*" or "data access" or "data transfer").mp. [mp=title, abstract, heading word, drug trade name, original title, device manufacturer, drug manufacturer, device trade name, keyword heading word, floating subheading word, candidate term word]

**4** *research/ or "precision medicine".mp. or "personal* medicine".mp. or "tailored medicine".mp. [mp=title, abstract, heading word, drug trade name, original title, device manufacturer, drug manufacturer, device trade name, keyword heading word, floating subheading word, candidate term word]

**5** 1 and 2 and 3 and 4

**6** limit 5 to (human and english language and yr="2012 -Current")

**Web of Science**

ALL=(patient* OR public OR citizen* OR lay) AND ALL=(attitude* OR view* OR perspective* OR opinion* OR trust) AND ALL=("data shar*" OR "data access" OR "data transfer") AND ALL=(research OR "secondary research" OR "precision medicine" OR "personal* medicine" OR "tailored medicine")
