# Supplementary material for: Stakeholders’ perceptions of personal health data sharing: A scoping review
Source: PLOS Digit Health. 2024 Nov 20;3(11):e0000652. doi: 10.1371/journal.pdig.0000652 (PMC11578505; doi:10.1371/journal.pdig.0000652)
Supplement: S3 Appendix — (DOCX) [file pdig.0000652.s003.docx]

**S3 Appendix: Coding framework for data extraction**

- Title
- Author
- Year
- Type of paper
- Journal
- Aim of research
- Research approach
- Data collection method
- Date of data collection
- Setting
- Sample size
- Sample characteristics
- Perspective
- Source of data studied
- Purpose of data sharing
- Perceptions and attitudes around data sharing
- Overall willingness to share data
- Motivations to share data
- Perceived benefits of sharing data
- Perceived risk of data sharing
- Awareness /understanding and uptake of technology
- Other barriers and facilitators to share data
